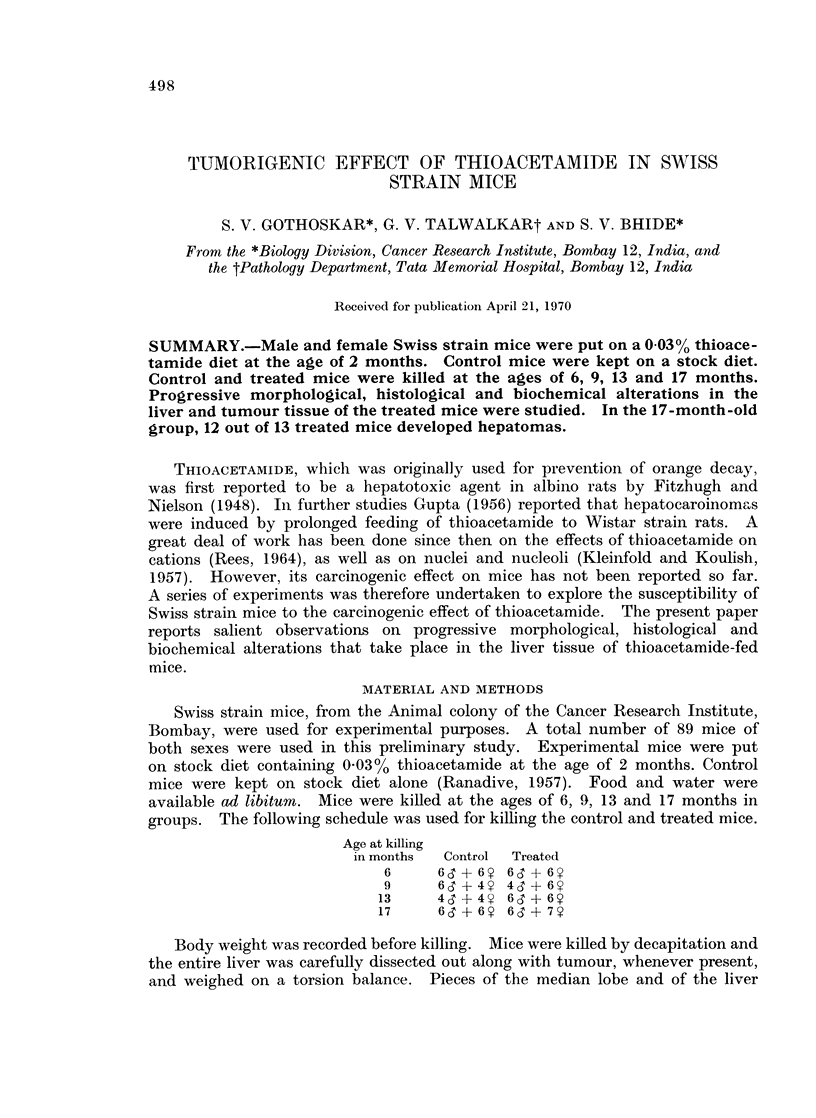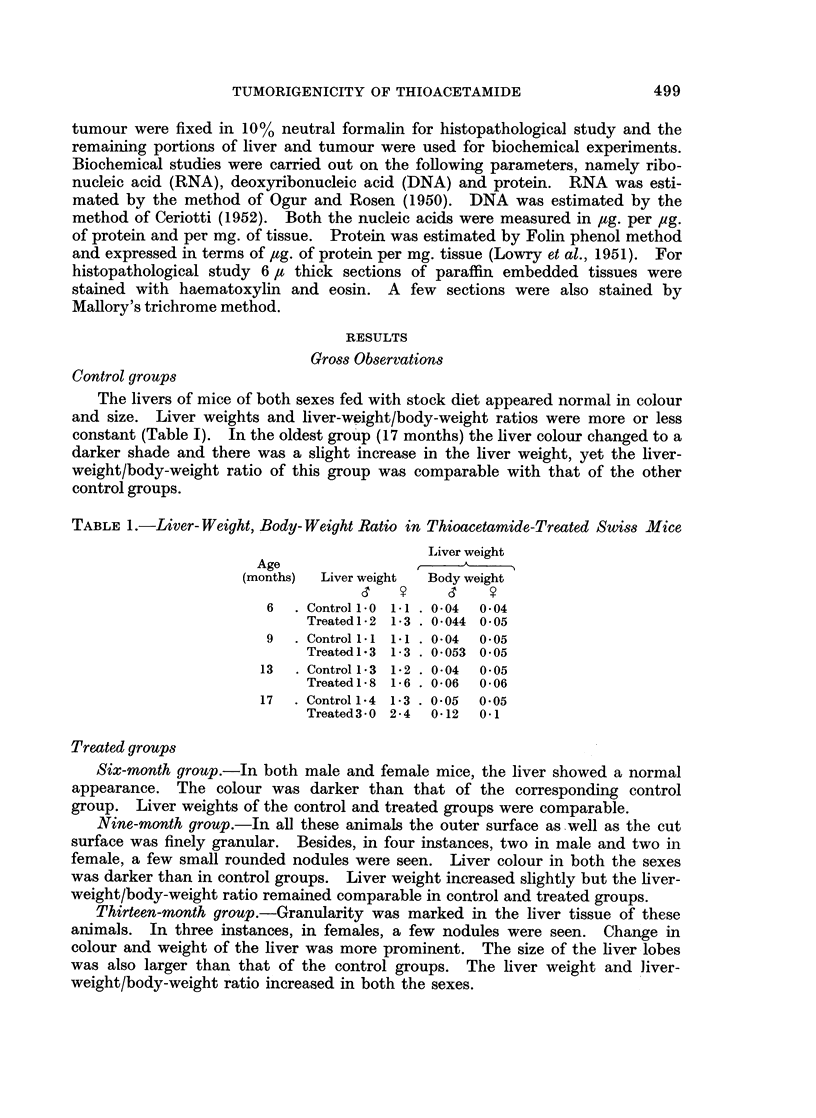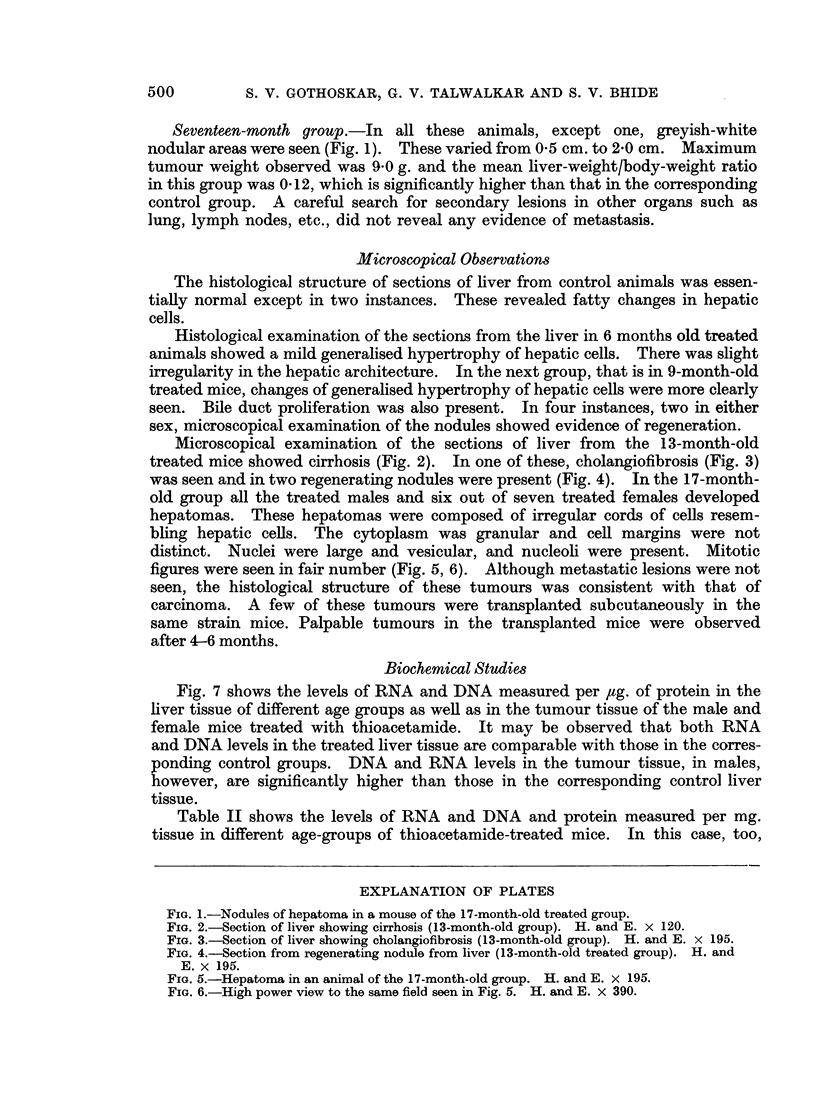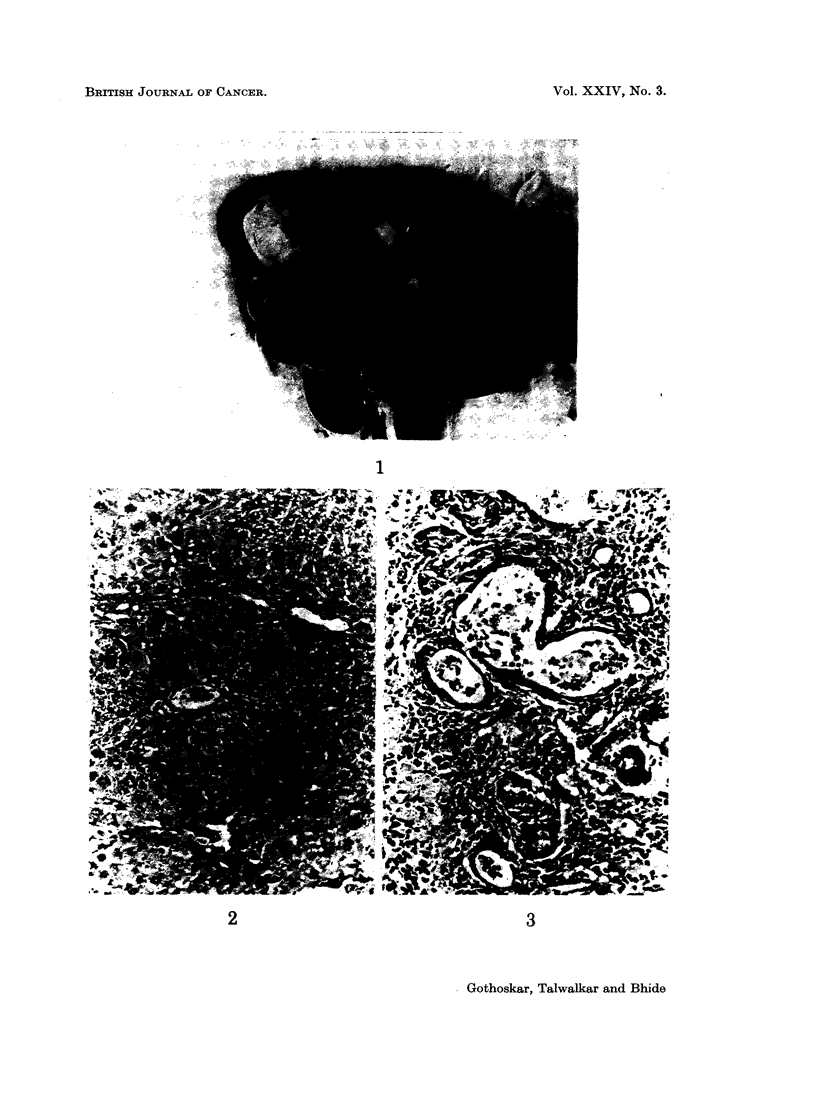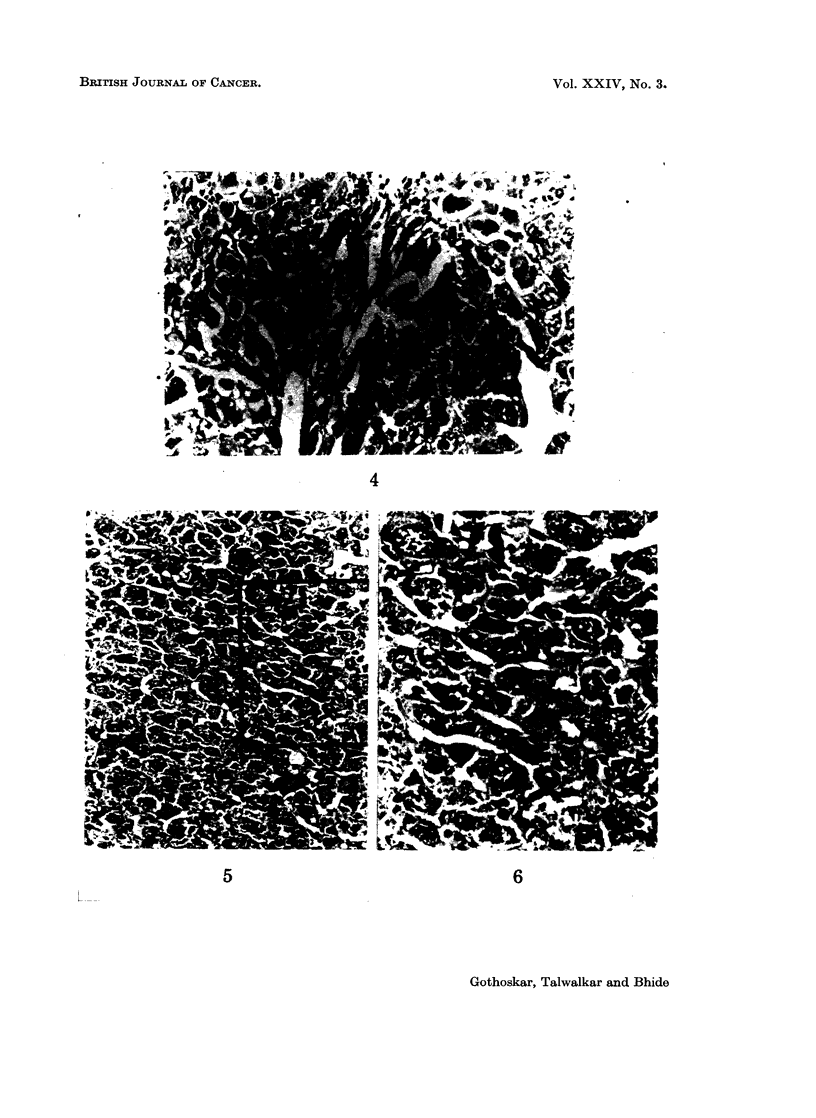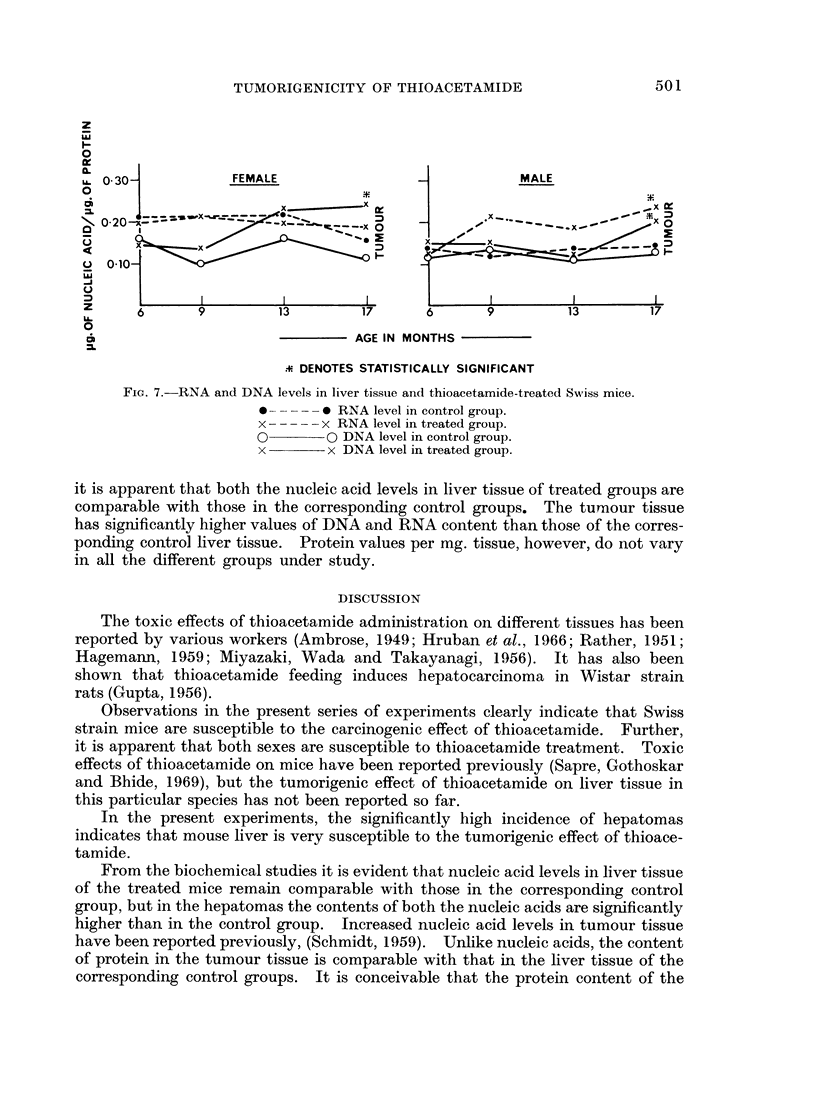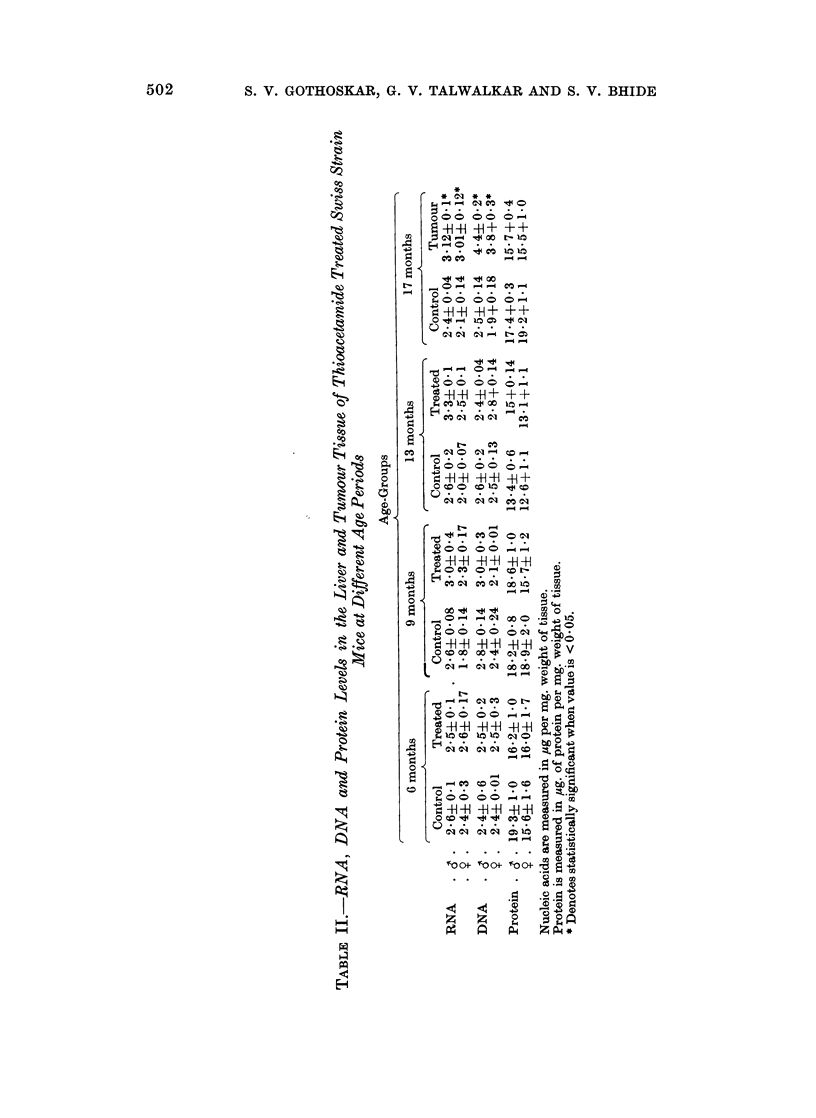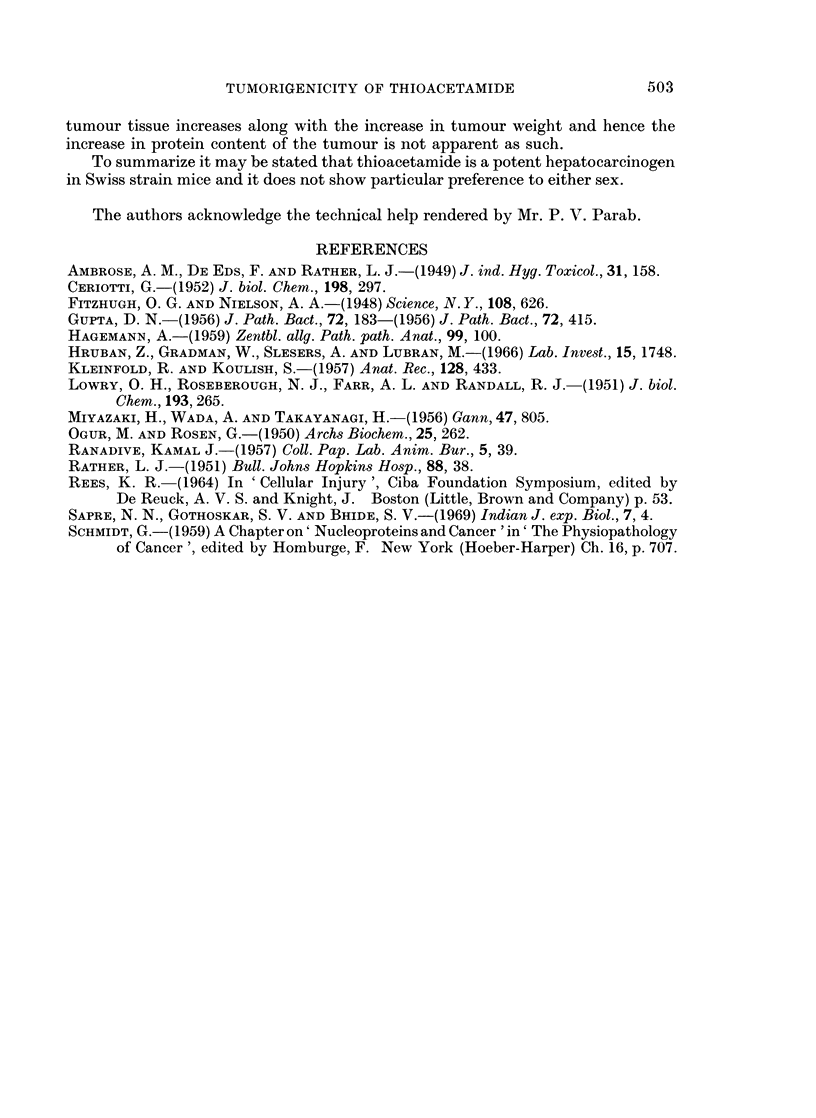# Tumorigenic Effect of Thioacetamide in Swiss Strain Mice

**DOI:** 10.1038/bjc.1970.60

**Published:** 1970-09

**Authors:** S. V. Gothoskar, G. V. Talwalkar, S. V. Bhide

## Abstract

**Images:**


### TUMORIGENIC EFFECT OF THIOACETAMIDE IN SWISS STRAIN MICE

S. V. GOTHOSKAR*, G. V. TALWALKAR† AND S. V. BHIDE*

*From the *Biology Division, Cancer Research Institute, Bombay 12, India, and the †Pathology Department, Tata Memorial Hospital, Bombay 12, India*

Received for publication April 21, 1970

**SUMMARY.—Male and female Swiss strain mice were put on a 0·03% thioacetamide diet at the age of 2 months. Control mice were kept on a stock diet. Control and treated mice were killed at the ages of 6, 9, 13 and 17 months. Progressive morphological, histological and biochemical alterations in the liver and tumour tissue of the treated mice were studied. In the 17-month-old group, 12 out of 13 treated mice developed hepatomas.**

THIOACETAMIDE, which was originally used for prevention of orange decay, was first reported to be a hepatotoxic agent in albino rats by Fitzhugh and Nielson (1948). In further studies Gupta (1956) reported that hepatocaroinomas were induced by prolonged feeding of thioacetamide to Wistar strain rats. A great deal of work has been done since then on the effects of thioacetamide on cations (Rees, 1964), as well as on nuclei and nucleoli (Kleinfold and Koulish, 1957). However, its carcinogenic effect on mice has not been reported so far. A series of experiments was therefore undertaken to explore the susceptibility of Swiss strain mice to the carcinogenic effect of thioacetamide. The present paper reports salient observations on progressive morphological, histological and biochemical alterations that take place in the liver tissue of thioacetamide-fed mice.

### MATERIAL AND METHODS

Swiss strain mice, from the Animal colony of the Cancer Research Institute, Bombay, were used for experimental purposes. A total number of 89 mice of both sexes were used in this preliminary study. Experimental mice were put on stock diet containing 0·03% thioacetamide at the age of 2 months. Control mice were kept on stock diet alone (Ranadive, 1957). Food and water were available *ad libitum*. Mice were killed at the ages of 6, 9, 13 and 17 months in groups. The following schedule was used for killing the control and treated mice.

| Age at killing in months | Control | Treated |
|---|---|---|
| 6 | 6♂ + 6♀ | 6♂ + 6♀ |
| 9 | 6♂ + 4♀ | 4♂ + 6♀ |
| 13 | 4♂ + 4♀ | 6♂ + 6♀ |
| 17 | 6♂ + 6♀ | 6♂ + 7♀ |

Body weight was recorded before killing. Mice were killed by decapitation and the entire liver was carefully dissected out along with tumour, whenever present, and weighed on a torsion balance. Pieces of the median lobe and of the liver tumour were fixed in 10% neutral formalin for histopathological study and the remaining portions of liver and tumour were used for biochemical experiments. Biochemical studies were carried out on the following parameters, namely ribonucleic acid (RNA), deoxyribonucleic acid (DNA) and protein. RNA was estimated by the method of Ogur and Rosen (1950). DNA was estimated by the method of Ceriotti (1952). Both the nucleic acids were measured in $\mu$g. per $\mu$g. of protein and per mg. of tissue. Protein was estimated by Folin phenol method and expressed in terms of $\mu$g. of protein per mg. tissue (Lowry *et al.*, 1951). For histopathological study 6 $\mu$ thick sections of paraffin embedded tissues were stained with haematoxylin and eosin. A few sections were also stained by Mallory's trichrome method.

### RESULTS

### *Gross Observations*

*Control groups*

The livers of mice of both sexes fed with stock diet appeared normal in colour and size. Liver weights and liver-weight/body-weight ratios were more or less constant (Table I). In the oldest group (17 months) the liver colour changed to a darker shade and there was a slight increase in the liver weight, yet the liver-weight/body-weight ratio of this group was comparable with that of the other control groups.

TABLE 1.—*Liver-Weight, Body-Weight Ratio in Thioacetamide-Treated Swiss Mice*

| Age (months) | | Liver weight ♂ | Liver weight ♀ | Liver weight / Body weight ♂ | Liver weight / Body weight ♀ |
|---|---|---|---|---|---|
| 6 | Control | 1·0 | 1·1 | 0·04 | 0·04 |
| | Treated | 1·2 | 1·3 | 0·044 | 0·05 |
| 9 | Control | 1·1 | 1·1 | 0·04 | 0·05 |
| | Treated | 1·3 | 1·3 | 0·053 | 0·05 |
| 13 | Control | 1·3 | 1·2 | 0·04 | 0·05 |
| | Treated | 1·8 | 1·6 | 0·06 | 0·06 |
| 17 | Control | 1·4 | 1·3 | 0·05 | 0·05 |
| | Treated | 3·0 | 2·4 | 0·12 | 0·1 |

*Treated groups*

*Six-month group.*—In both male and female mice, the liver showed a normal appearance. The colour was darker than that of the corresponding control group. Liver weights of the control and treated groups were comparable.

*Nine-month group.*—In all these animals the outer surface as well as the cut surface was finely granular. Besides, in four instances, two in male and two in female, a few small rounded nodules were seen. Liver colour in both the sexes was darker than in control groups. Liver weight increased slightly but the liver-weight/body-weight ratio remained comparable in control and treated groups.

*Thirteen-month group.*—Granularity was marked in the liver tissue of these animals. In three instances, in females, a few nodules were seen. Change in colour and weight of the liver was more prominent. The size of the liver lobes was also larger than that of the control groups. The liver weight and liver-weight/body-weight ratio increased in both the sexes.

*Seventeen-month group.*—In all these animals, except one, greyish-white nodular areas were seen (Fig. 1). These varied from 0·5 cm. to 2·0 cm. Maximum tumour weight observed was 9·0 g. and the mean liver-weight/body-weight ratio in this group was 0·12, which is significantly higher than that in the corresponding control group. A careful search for secondary lesions in other organs such as lung, lymph nodes, etc., did not reveal any evidence of metastasis.

### *Microscopical Observations*

The histological structure of sections of liver from control animals was essentially normal except in two instances. These revealed fatty changes in hepatic cells.

Histological examination of the sections from the liver in 6 months old treated animals showed a mild generalised hypertrophy of hepatic cells. There was slight irregularity in the hepatic architecture. In the next group, that is in 9-month-old treated mice, changes of generalised hypertrophy of hepatic cells were more clearly seen. Bile duct proliferation was also present. In four instances, two in either sex, microscopical examination of the nodules showed evidence of regeneration.

Microscopical examination of the sections of liver from the 13-month-old treated mice showed cirrhosis (Fig. 2). In one of these, cholangiofibrosis (Fig. 3) was seen and in two regenerating nodules were present (Fig. 4). In the 17-month-old group all the treated males and six out of seven treated females developed hepatomas. These hepatomas were composed of irregular cords of cells resembling hepatic cells. The cytoplasm was granular and cell margins were not distinct. Nuclei were large and vesicular, and nucleoli were present. Mitotic figures were seen in fair number (Fig. 5, 6). Although metastatic lesions were not seen, the histological structure of these tumours was consistent with that of carcinoma. A few of these tumours were transplanted subcutaneously in the same strain mice. Palpable tumours in the transplanted mice were observed after 4–6 months.

### *Biochemical Studies*

Fig. 7 shows the levels of RNA and DNA measured per $\mu$g. of protein in the liver tissue of different age groups as well as in the tumour tissue of the male and female mice treated with thioacetamide. It may be observed that both RNA and DNA levels in the treated liver tissue are comparable with those in the corresponding control groups. DNA and RNA levels in the tumour tissue, in males, however, are significantly higher than those in the corresponding control liver tissue.

Table II shows the levels of RNA and DNA and protein measured per mg. tissue in different age-groups of thioacetamide-treated mice. In this case, too,

---

EXPLANATION OF PLATES

FIG. 1.—Nodules of hepatoma in a mouse of the 17-month-old treated group.
FIG. 2.—Section of liver showing cirrhosis (13-month-old group). H. and E. × 120.
FIG. 3.—Section of liver showing cholangiofibrosis (13-month-old group). H. and E. × 195.
FIG. 4.—Section from regenerating nodule from liver (13-month-old treated group). H. and E. × 195.
FIG. 5.—Hepatoma in an animal of the 17-month-old group. H. and E. × 195.
FIG. 6.—High power view to the same field seen in Fig. 5. H. and E. × 390.

1

2

3

Gothoskar, Talwalkar and Bhide

4

5

6

Gothoskar, Talwalkar and Bhide

FIG. 7.—RNA and DNA levels in liver tissue and thioacetamide-treated Swiss mice.

●– – – – – –● RNA level in control group.

×– – – – – –× RNA level in treated group.

○————○ DNA level in control group.

×————× DNA level in treated group.

it is apparent that both the nucleic acid levels in liver tissue of treated groups are comparable with those in the corresponding control groups. The tumour tissue has significantly higher values of DNA and RNA content than those of the corresponding control liver tissue. Protein values per mg. tissue, however, do not vary in all the different groups under study.

### DISCUSSION

The toxic effects of thioacetamide administration on different tissues has been reported by various workers (Ambrose, 1949; Hruban *et al.*, 1966; Rather, 1951; Hagemann, 1959; Miyazaki, Wada and Takayanagi, 1956). It has also been shown that thioacetamide feeding induces hepatocarcinoma in Wistar strain rats (Gupta, 1956).

Observations in the present series of experiments clearly indicate that Swiss strain mice are susceptible to the carcinogenic effect of thioacetamide. Further, it is apparent that both sexes are susceptible to thioacetamide treatment. Toxic effects of thioacetamide on mice have been reported previously (Sapre, Gothoskar and Bhide, 1969), but the tumorigenic effect of thioacetamide on liver tissue in this particular species has not been reported so far.

In the present experiments, the significantly high incidence of hepatomas indicates that mouse liver is very susceptible to the tumorigenic effect of thioacetamide.

From the biochemical studies it is evident that nucleic acid levels in liver tissue of the treated mice remain comparable with those in the corresponding control group, but in the hepatomas the contents of both the nucleic acids are significantly higher than in the control group. Increased nucleic acid levels in tumour tissue have been reported previously, (Schmidt, 1959). Unlike nucleic acids, the content of protein in the tumour tissue is comparable with that in the liver tissue of the corresponding control groups. It is conceivable that the protein content of the

TABLE II.—*RNA, DNA and Protein Levels in the Liver and Tumour Tissue of Thioacetamide Treated Swiss Strain Mice at Different Age Periods*

| | | Age-Groups | | | | | | | |
|---|---|---|---|---|---|---|---|---|---|
| | | 6 months | | 9 months | | 13 months | | 17 months | |
| | | Control | Treated | Control | Treated | Control | Treated | Control | Tumour |
| RNA | ♂ | 2·6±0·1 | 2·5±0·1 | 2·6±0·08 | 3·0±0·4 | 2·6±0·2 | 3·3±0·1 | 2·4±0·04 | 3·12±0·1* |
| | ♀ | 2·4±0·3 | 2·6±0·17 | 1·8±0·14 | 2·3±0·17 | 2·0±0·07 | 2·5±0·1 | 2·1±0·14 | 3·01±0·12* |
| DNA | ♂ | 2·4±0·6 | 2·5±0·2 | 2·8±0·14 | 3·0±0·3 | 2·6±0·2 | 2·4±0·04 | 2·5±0·14 | 4·4±0·2* |
| | ♀ | 2·4±0·01 | 2·5±0·3 | 2·4±0·24 | 2·1±0·01 | 2·5±0·13 | 2·8+0·14 | 1·9+0·18 | 3·8+0·3* |
| Protein | ♂ | 19·3±1·0 | 16·2±1·0 | 18·2±0·8 | 18·6±1·0 | 13·4±0·6 | 15+0·14 | 17·4+0·3 | 15·7+0·4 |
| | ♀ | 15·6±1·6 | 16·0±1·7 | 18·9±2·0 | 15·7±1·2 | 12·6+1·1 | 13·1+1·1 | 19·2+1·1 | 15·5+1·0 |

Nucleic acids are measured in μg per mg. weight of tissue.

Protein is measured in μg. of protein per mg. weight of tissue.

* Denotes statistically significant when value is < 0·05.

tumour tissue increases along with the increase in tumour weight and hence the increase in protein content of the tumour is not apparent as such.

To summarize it may be stated that thioacetamide is a potent hepatocarcinogen in Swiss strain mice and it does not show particular preference to either sex.

The authors acknowledge the technical help rendered by Mr. P. V. Parab.

### REFERENCES

AMBROSE, A. M., DE EDS, F. AND RATHER, L. J.—(1949) *J. ind. Hyg. Toxicol.*, **31**, 158.
CERIOTTI, G.—(1952) *J. biol. Chem.*, **198**, 297.
FITZHUGH, O. G. AND NIELSON, A. A.—(1948) *Science, N.Y.*, **108**, 626.
GUPTA, D. N.—(1956) *J. Path. Bact.*, **72**, 183—(1956) *J. Path. Bact.*, **72**, 415.
HAGEMANN, A.—(1959) *Zentbl. allg. Path. path. Anat.*, **99**, 100.
HRUBAN, Z., GRADMAN, W., SLESERS, A. AND LUBRAN, M.—(1966) *Lab. Invest.*, **15**, 1748.
KLEINFOLD, R. AND KOULISH, S.—(1957) *Anat. Rec.*, **128**, 433.
LOWRY, O. H., ROSEBEROUGH, N. J., FARR, A. L. AND RANDALL, R. J.—(1951) *J. biol. Chem.*, **193**, 265.
MIYAZAKI, H., WADA, A. AND TAKAYANAGI, H.—(1956) *Gann*, **47**, 805.
OGUR, M. AND ROSEN, G.—(1950) *Archs Biochem.*, **25**, 262.
RANADIVE, KAMAL J.—(1957) *Coll. Pap. Lab. Anim. Bur.*, **5**, 39.
RATHER, L. J.—(1951) *Bull. Johns Hopkins Hosp.*, **88**, 38.
REES, K. R.—(1964) In ‘ Cellular Injury ’, Ciba Foundation Symposium, edited by De Reuck, A. V. S. and Knight, J. Boston (Little, Brown and Company) p. 53.
SAPRE, N. N., GOTHOSKAR, S. V. AND BHIDE, S. V.—(1969) *Indian J. exp. Biol.*, **7**, 4.
SCHMIDT, G.—(1959) A Chapter on ‘ Nucleoproteins and Cancer ’ in ‘ The Physiopathology of Cancer ’, edited by Homburge, F. New York (Hoeber-Harper) Ch. 16, p. 707.